# Metagenomics insights into food fermentations

**DOI:** 10.1111/1751-7915.12421

**Published:** 2016-10-06

**Authors:** Francesca De Filippis, Eugenio Parente, Danilo Ercolini

**Affiliations:** ^1^ Division of MicrobiologyDepartment of Agricultural SciencesUniversity of Naples Federico IIPorticiItaly; ^2^ Dipartimento di ScienzeUniversità degli Studi della BasilicataPotenzaItaly

## Abstract

This review describes the recent advances in the study of food microbial ecology, with a focus on food fermentations. High‐throughput sequencing (HTS) technologies have been widely applied to the study of food microbial *consortia* and the different applications of HTS technologies were exploited in order to monitor microbial dynamics in food fermentative processes. Phylobiomics was the most explored application in the past decade. Metagenomics and metatranscriptomics, although still underexploited, promise to uncover the functionality of complex microbial *consortia*. The new knowledge acquired will help to understand how to make a profitable use of microbial genetic resources and modulate key activities of beneficial microbes in order to ensure process efficiency, product quality and safety.

## Introduction

Culture‐independent techniques have helped to change the way to study food microbial ecology, leading to consider microbial populations as *consortia* (Cocolin and Ercolini, 2015). Further evolution was stimulated by the advent of high‐throughput sequencing (HTS) technologies in the mid 2000s, which in the past decade became ubiquitous in microbial ecology studies. HTS entails higher sensitivity compared with traditional culture‐independent methods beyond being considered quantitative. Two different HTS approaches can be used. In the most commonly applied option, marker‐genes are amplified from genomic DNA (or RNA, after a reverse‐transcription step) through PCR and sequenced. Taxonomic relevant genes are usually sequenced through this approach, leading to the description of the phylobiome (van Hijum *et al*., 2013), that is the taxonomic composition of the microbial community and the relative abundance of its members. In metagenomics or metatranscriptomics studies, no PCR is performed and total DNA or cDNA is sequenced. Besides the taxonomical composition of the community, this approach allows obtaining the abundance of all microbial genes. The output of metagenomics analysis, based on DNA sequencing, are the potential activities of the microbial communities, because a specific gene may not be really expressed in that condition or because DNA may arise from dead or metabolically inactive cells. In order to identify the genes actually expressed in a food sample, RNA sequencing (RNA‐seq) is the most appropriate path to take, which is what happens in metatranscriptomics.

The study of microbial ecology is relevant in biotechnology as it is the basis for not only the development of fermentations but also for the comprehension of the microbial interactions that drive a premium quality process. The availability of such a powerful tool box offers tantalizing opportunities to study food microbes understanding how their potential functions can be changed or modulated with the ultimate scope of improving food quality.

## Monitoring microbes in food fermentations

**Figure 1:**
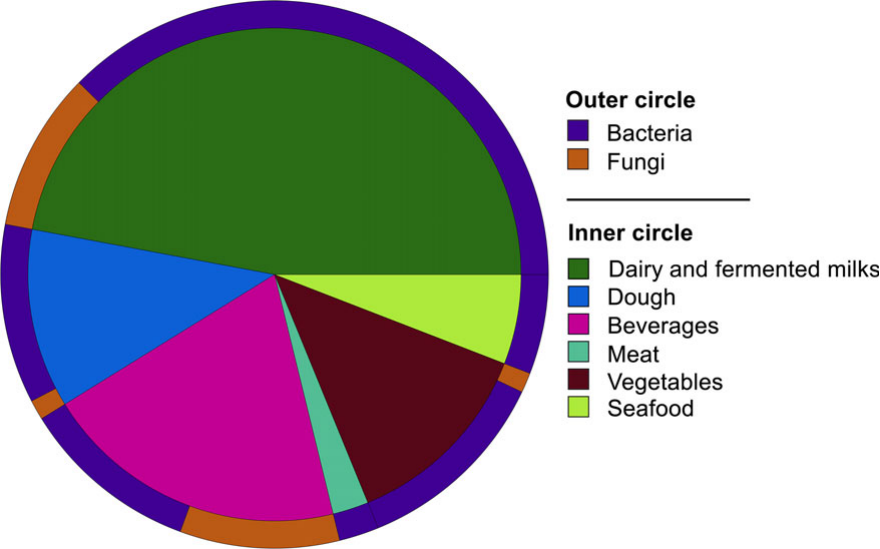
Pie chart showing the abundance of HTS studies of fermented foods and beverages grouped according to the food matrix. For each food environment, the outer circle shows the proportion of studies analysing bacterial or fungal communities.

Amplicon‐based HTS targeting genes of taxonomic relevance has become the most widely exploited approach in food microbial ecology. In the past decade, it was widely used to monitor microbial communities during fermentation of different types of foodstuffs and beverages (Fig. 1). Table 1 reports an extensive, although not complete, list of them. Several questions can be addressed by the description of microbial communities during fermentations. An in‐depth characterization of the normal or abnormal microbial *consortia* at different stages of fermentations is important in order to evaluate lot‐to‐lot consistency, identify biomarkers for product quality or spoilage, and learning how to manipulate fermentation conditions to improve the process control.

**Table 1:** HTS studies of food fermentations. Studies are grouped according to the type of food and ordered by year of publication

Target gene	Short description	Year	Food Group	Reference
16S rRNA gene (Bacteria)	Kefir grains and kefir milk	2011	Dairy and fermented milks	Dobson et al. (2011)
16S rRNA gene (Bacteria)	Danish raw milk cheese during ripening	2011	Dairy and fermented milks	Masoud et al. (2011)
16S rRNA gene (Bacteria)	Kefir grains from different parts of Brazil	2012	Dairy and fermented milks	Leite et al. (2012)
16S rRNA gene (Bacteria)	Mozzarella cheese (Italy) and intermediates from two manufactures	2012	Dairy and fermented milks	Ercolini et al. (2012)
16S rRNA gene (Bacteria)	Latin style cheese	2012	Dairy and fermented milks	Lusk et al. (2012)
16S rRNA gene (Bacteria)	Curd, fresh and smoked Polish cheese (Oscypek)	2012	Dairy and fermented milks	Alegria et al. (2012)
16S rRNA gene (Bacteria)	Artisanal soft, semi‐hard and hard cheeses from raw or pasteurized cow, goat, or sheep milk	2012	Dairy and fermented milks	Quigley et al. (2012)
16S rRNA gene (Bacteria); ITS1‐2 (Fungi)	Kefir grain and kefir milk from different sources	2013	Dairy and fermented milks	Marsh et al. (2013)
16S rRNA gene (Bacteria); ITS1‐2 (Fungi)	Swabs from cheesemaking environment and cheese	2013	Dairy and fermented milks	Bokulich et al. (2013a)
16S rRNA gene (Bacteria)	Croatiam raw ewe's milk cheese during ripening	2013	Dairy and fermented milks	Fuka et al. (2013)
16S rRNA gene (Bacteria)	Turkish kefir grains	2014	Dairy and fermented milks	Nalbantoglu et al. (2014)
16S rRNA gene (Bacteria)	Whey cultures and cheese curds from water‐buffalo Mozzarella, Grana Padano and Parmigiano Reggiano cheese (Italy) manufacturing	2014	Dairy and fermented milks	De Filippis et al. (2014)
16S rRNA gene (Bacteria)	Ewe milk, curd and Canestrato cheese (Italy) during ripening	2014	Dairy and fermented milks	De Pasquale et al. (2014a)
16S rRNA gene (Bacteria)	Cow milk, curd and Caciocavallo cheese (Italy) during ripening	2014	Dairy and fermented milks	De Pasquale et al. (2014b)
16S rRNA gene (Bacteria)	Cow milk (from different lactation stages), curd and Fontina cheese (Italy) from three dairies	2014	Dairy and fermented milks	Dolci et al. (2014)
16S rRNA gene (Bacteria); ITS1‐2 (Fungi)	Bloomy, natural and washed cheese rinds	2014	Dairy and fermented milks	Wolfe et al. (2014)
16S rRNA gene (Bacteria)	Traditional Pico cheese (Portugal) manufactured in three different dairies, monitored during ripening	2014	Dairy and fermented milks	Riquelme et al. (2014)
16S rRNA gene (Bacteria)	Samples of milk, whey, curd and ripened Poro cheese (Mexico)	2014	Dairy and fermented milks	Aldrete‐Tapia et al. (2014)
16S rRNA gene (Bacteria); ITS1‐2 (Fungi)	Tarag (fermented dairy product) from China and Mongolia	2014	Dairy and fermented milks	Sun et al. (2014)
16S rRNA gene (Bacteria)	Samples of core and rind of Herve cheese (Belgium)	2014	Dairy and fermented milks	Delcenserie et al. (2014)
16S rRNA gene (Bacteria)	Chinese traditional fermented milk (yond bap) from cow or goat milk	2015	Dairy and fermented milks	Liu et al. (2015a)
16S rRNA gene (Bacteria); 18S rRNA gene (Fungi)	Naturally fermented cow milks from Mongolia	2015	Dairy and fermented milks	Liu et al. (2015b)
16S rRNA gene (Bacteria); 26S rRNA gene (Fungi)	Milk kefir grains from different Italian regions	2015	Dairy and fermented milks	Garofalo et al. (2015)
16S rRNA gene (Bacteria); ITS1‐2 (Fungi)	Matsoni (fermented milk) samples from several geographic areas	2015	Dairy and fermented milks	Bokulich et al. (2015a)
16S rRNA gene (Bacteria); 26S rRNA gene (Fungi)	Environmental swabs from a dairy plant and cheeses (Italy)	2015	Dairy and fermented milks	Stellato et al. (2015)
16S rRNA gene (Bacteria)	Continental cheese produced early and late in the day, at different ripening times	2015	Dairy and fermented milks	O'Sullivan et al. (2015)
16S rRNA gene (Bacteria)	Commercial high‐moisture Mozzarella cheese produced with different acidification methods	2016	Dairy and fermented milks	Guidone et al. (2015)
16S rRNA gene (Bacteria)	Undefined strain starters (milk cultures) for high‐moisture Mozzarella cheese	2016	Dairy and fermented milks	Parente et al. (2016b)
16S rRNA gene (Bacteria)	Grana‐type cheese (Italy) during ripening	2016	Dairy and fermented milks	Alessandria et al. (2016)
16S rRNA gene (Bacteria)	Natural whey culture, milk, curd and Caciocavallo cheese (Italy) during ripening	2016	Dairy and fermented milks	De Filippis et al. (2016a)
16S rRNA gene (Bacteria)	Environmental swabs from a dairy plant and cheeses (Italy)	2016	Dairy and fermented milks	Calasso et al. (2016)
16S rRNA gene (Bacteria)	Spatial distribution of microbiota in Italian ewes’ milk cheese	2016	Dairy and fermented milks	De Pasquale et al. (2016)
16S rRNA gene (Bacteria)	Rye, durum and common wheat sourdough	2013	Doughs	Ercolini et al. (2013)
16S rRNA gene (Bacteria)	Traditional sweet leavened doughs	2013	Doughs	Lattanzi et al. (2013)
16S rRNA gene (Bacteria)	Rye sourdoughs propagated for two months at 20 and 30°C	2014	Doughs	Bessmeltseva et al. (2014)
16S rRNA gene (Bacteria)	Flour and sourdough made of durum wheat grown under organic and conventional farming	2015	Doughs	Rizzello et al. (2015)
16S rRNA gene (Bacteria); 18S rRNA gene (Fungi)	Flour, doughs and related food environments	2015	Doughs	Minervini et al. (2015)
16S rRNA gene (Bacteria)	Wheat sourdoughs used for traditional breads in different regions of France	2015	Doughs	Lhomme et al. (2015a)
16S rRNA gene (Bacteria)	Sourdoughs used for the manufacture of traditional French breads	2015	Doughs	Lhomme et al. (2015b)
16S rRNA gene (Bacteria)	Dough samples during manufacture of Chica (a fermented maize product) in Argentina	2015	Doughs	Elizaquível et al. (2015)
16S rRNA gene (Bacteria)	Rye sourdoughs from four Estonian bakeries	2016	Doughs	Viiard et al. (2016)
16S rRNA gene (Bacteria)	Botrityzed wine during fermentation, three vintages, inoculated and uninoculated bactches	2012	Fermented beverages ‐ grapes	Bokulich et al. (2012a)
16S rRNA gene (Bacteria); ITS1‐2 (Fungi)	Grape must samples collected in California over two different vintages	2014	Fermented beverages ‐ grapes	Bokulich et al. (2014a)
16S rRNA gene (Bacteria); 26S rRNA gene and ITS1‐2 (Fungi)	Grape must samples collected in different Portuguese regions during fermentation	2015	Fermented beverages ‐ grapes	Pinto et al. (2015)
26S rRNA gene (Fungi)	Spanish grape must samples during fermentation	2015	Fermented beverages ‐ grapes	Wang et al. (2015)
18S rRNA gene (Fungi)	Italian traditional wine fermentations	2016	Fermented beverages ‐ grapes	De Filippis et al. (2016b)
16S rRNA gene (Bacteria); ITS1‐2 (Fungi)	Grape must samples collected in three wineries in Northern Italy during fermentation	2016	Fermented beverages ‐ grapes	Stefanini et al. (2016)
16S rRNA gene (Bacteria)	Beer (American Coolship Ale) during fermentation and related environment	2012	Fermented beverages ‐ malt	Bokulich et al. (2012b)
16S rRNA gene (Bacteria)	Barley during malting, two different seasons	2014	Fermented beverages ‐ malt	Justé et al. (2014)
16S rRNA gene (Bacteria); ITS1‐2 (Fungi)	Traditional Korean alcoholic beverage (Makgeolli), and the starter (Nuruk), during fermentation	2012	Fermented beverages ‐ rice	Jung et al. (2012)
16S rRNA gene (Bacteria); ITS1‐2 (Fungi)	Kimoto sake during manufacturing and related environmental samples	2014	Fermented beverages ‐ rice	Bokulich et al. (2014b)
16S rRNA gene (Bacteria); ITS1‐2 (Fungi)	Tea fungus (kombucha) samples during fermentation	2014	Fermented beverages ‐ tea	Marsh et al. (2014)
16S rRNA gene (Bacteria); ITS1‐4 (Fungi)	Pu‐erh Japanese traditional tea during fermentation	2015	Fermented beverages ‐ tea	Zhao et al. (2015)
16S rRNA gene (Bacteria)	Fermented meat (salami) during ripening	2015	Meat	Greppi et al. (2015)
16S rRNA gene (Bacteria)	Fermented meat (salami) during ripening	2015	Meat	Polka et al. (2015)
16S rRNA gene (Bacteria)	Fermented seafood	2010	Seafood	Roh et al. (2010)
16S rRNA gene (Bacteria)	Narezushi (salted and fermented fish, rice, peppers)	2011	Seafood	Koyanagi et al. (2011)
16S rRNA gene (Bacteria)	Traditional fermented sushi (kaburazushi),during fermentation	2013	Seafood	Koyanagi et al. (2013)
16S rRNA gene (Bacteria)	Traditional fermented shrimp (Saeu‐jeot) during fermentation	2013	Seafood	Jung et al. (2013)
16S rRNA gene (Bacteria)	Traditional fermented shrimp (Saeu‐jeot) during fermentation at different temperatures	2014	Seafood	Lee et al. (2014)
16S rRNA gene (Bacteria)	Korean fish sauce (Myeolchi‐Aekjeot)	2015	Seafood	Lee et al. (2015)
16S rRNA gene (Bacteria)	Pearl millet slurried with or without groundnuts at the beginning and end of fermentation	2009	Vegetables	Humblot et al. (2009)
16S rRNA gene (Bacteria)	Vegetable pickle from rice bran (Nukadoko)	2011	Vegetables	Sakamoto et al. (2011)
16S rRNA gene (Bacteria)	Baechu (Chinese cabbage) and Chonggak (radish) kimchi prepared with and without starter	2012	Vegetables	Jung et al. (2012)
16S rRNA gene (Bacteria)	Fermented Korean soybean paste (Doenjang)	2012	Vegetables	Nam et al. (2012a)
16S rRNA gene (Bacteria)	Traditional Korean fermented food (Kochujang) made of rice, pepper, soybeans	2012	Vegetables	Nam et al. (2012b)
16S rRNA gene (Bacteria)	Ten different varieties of kimchi, during fermentation	2012	Vegetables	Park et al. (2012)
16S rRNA gene (Bacteria)	Olive surfaces and brine during fermentation	2013	Vegetables	Cocolin et al. (2013)
16S rRNA gene (Bacteria)	Kimchi samples during fermentation (100 days)	2013	Vegetables	Jeong et al. (2013)
16S rRNA gene (Bacteria)	Fermented Korean soybean lumps (meju)	2014	Vegetables	Jung et al. (2014)
16S rRNA gene (Bacteria)	Started and unstarted Bella di Cerignola table olives	2015	Vegetables	De Angelis et al. (2015)

Dairy is by far the most explored environment (Fig. 1) and a broad variety of cheeses were studied through amplicon‐based HTS, allowing monitoring of curd fermentation (Ercolini *et al*., 2012; De Filippis *et al*., 2014) or cheese ripening (Fuka *et al*., 2013; De Pasquale *et al*., 2014b; O'Sullivan *et al*., 2015; Alessandria *et al*., 2016; De Filippis *et al*., 2016a) and exploring the spatial distribution of microbes in different parts of the same cheese (O'Sullivan *et al*., 2015; De Filippis *et al*., 2016a; De Pasquale *et al*., 2016).

In many cases, the study of food microbiota highlighted possible relationships between microbial community structure/dynamics and physicochemical parameters, such as pH, water activity (a_w_), salt concentration and temperature (Bessmeltseva *et al*., 2014; Wolfe *et al*., 2014; Lhomme *et al*., 2015a,b; Minervini *et al*., 2015; De Filippis *et al*., 2016a). In other studies, the microbiota was related to raw material origin (Bokulich *et al*., 2014a; Rizzello *et al*., 2015) or quality (Dolci *et al*., 2014; O'Sullivan *et al*., 2015; Alessandria *et al*., 2016), as well as to development of flavour‐impact compounds (De Pasquale *et al*., 2014a, 2016; De Filippis *et al*., 2016a). Moreover, food‐related environments were found to harbour a resident microbiota, beneficially involved in dairy (Bokulich and Mills, 2013a; Stellato *et al*., 2015; Calasso *et al*., 2016), alcoholic (Bokulich *et al*., 2012a, 2014b) and sourdough (Minervini *et al*., 2015) fermentations, although the presence of potential spoilers was also emphasized in some cases (Bokulich *et al*., 2015a; Stellato *et al*., 2015).

Although fungi can be very important in some kinds of food fermentations, there is a huge difference in the number of published studies describing fungal and bacterial communities through HTS (Fig. 1). While 16S ribosomal RNA (rRNA) is the common choice for bacteria, more variability in the target gene to use was highlighted for fungi (Table 1). The most used target is the internal transcribed spacer (ITS), most of all thanks to the presence of a well‐curated database (https://unite.ut.ee). However, the uneven ITS length among species may promote preferential amplification of shorter fragments during the PCR step and therefore higher number of sequences for those OTUs with shorter ITS fragment. Since the OTU abundance is proportional to the number of reads obtained, this may lead to an incorrect estimation of OTU abundance (Bokulich and Mills, 2013b; Ercolini, 2013; De Filippis and Ercolini, 2016). Therefore, the use of different targets would be also advisable, such as the 26S (Garofalo *et al*., 2015; Stellato *et al*., 2015; Wang *et al*., 2015) or the 18S rRNA (Liu *et al*., 2015b; Minervini *et al*., 2015) genes.

Although PCR‐dependent, the amplicon‐based HTS is considered quantitative: the number of reads obtained for each operational taxonomic unit (OTU) is proportional to the abundance of that OTU in the sample and the higher sensitivity allows identifying also sub‐populations previously difficult to detect. However, the data generated need to be interpreted with the awareness of culture‐independent PCR biases that have been reviewed elsewhere (Sipos *et al*., 2010), such as the possibility of preferential amplification, due to the different efficiency of the primer towards selected species, that may result in the under‐representation of some clades (Pinto and Raskin, 2012).

In several occasions RNA was preferred as target instead of DNA, that may arise from dead or inactive cells (Ercolini *et al*., 2013; Rizzello *et al*., 2015; De Filippis *et al*., 2016a). Since RNA is more easily degraded, it allows obtaining a structure of the microbiota that is potentially ascribable to the viable populations. Nevertheless, the treatment of food samples with propidium monoazide (PMA) was recently proposed as an alternative (Erkus *et al*., 2016) to the use of RNA templates to describe live microbial populations. PMA selectively binds to DNA freely present in the matrix, arising from dead or compromised cells, but cannot enter intact cell membranes. Therefore, treatment of the sample with PMA before DNA extraction inhibits its amplification by PCR and allows obtaining a profile of the viable microbial community (Erkus *et al*., 2016).

Notwithstanding the thick body of literature accumulated on food microbial communities assessed by amplicon sequencing, most of the studies are basically descriptive. In addition, well‐known microbial players have been identified and thus limited new information was provided on food fermentative processes.

**Figure 2:**
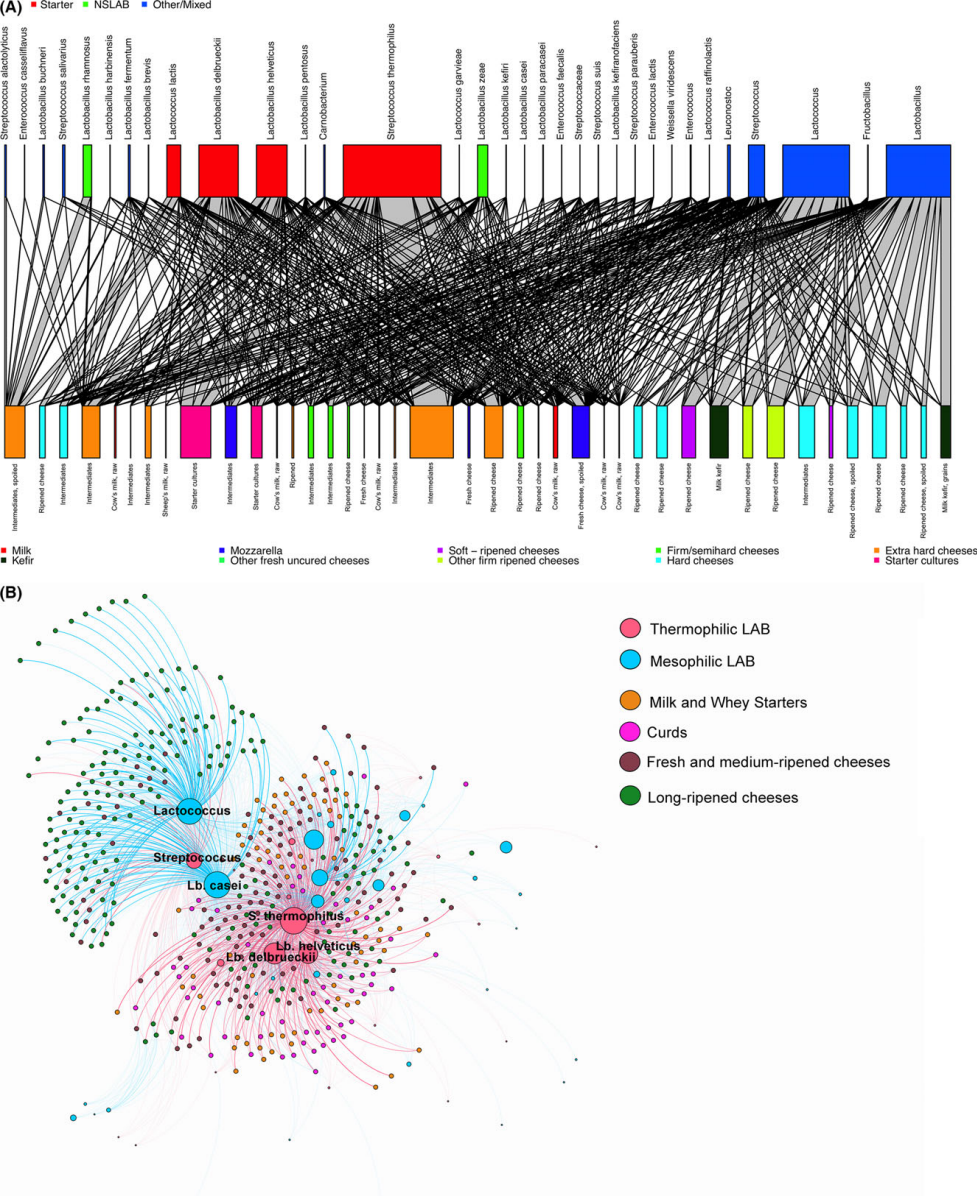
Graphical representations of the network structure of dairy samples extracted from Foodmicrobionet (v. 2.0). In (A), individual dairy samples are coulored according to the type of dairy product and aggregated by the state of the food (finished, intermediate, raw), the occurrence of spoilage, fermentation or ripening.

However, thanks to the extensive amount of 16S rRNA gene data generated by amplicon‐based food microbiota descriptions, there currently is an unprecedented possibility of data sharing. Sequence data can be made available through public databases, e.g. the Sequence Read Archive of the National Center for Biotechnology Information (http://www.ncbi.nlm.nih.gov/Traces/sra) or the European Nucleotide Archive of the European Bioinformatics Institute (http://www.ebi.ac.uk/ena). In this way, researchers from all over the world can easily access datasets generated by other laboratories, re‐analyse and use them for meta‐studies. FoodMicrobionet (http://www.foodmicrobionet.org, Parente *et al*., 2016a) was recently launched with the aim to collect the results obtained in different HTS studies of food microbial ecology and to provide an easy‐to‐use tool for visualization and comparison of the microbiota in diverse foodstuffs. Samples are classified using the FoodEx classification, as suggested by the European Food Safety Authority (http://www.efsa.europa.eu/en/data/data-standardisation). Further metadata include the nature of food (raw, intermediate, finished), the use of thermal treatments and the occurrence of spoilage and/or fermentation. Users can easily extract subsets of samples for the food matrix of interest, recreate abundance tables in long and wide format and visualize them in a network or use in comparative studies. As an example, graphical representations of the network structure of dairy samples extracted from Foodmicrobionet (v. 2.0) are provided here (Fig. 2). Figure 2A, a bipartite network representation, shows high abundance of starter lactic acid bacteria (SLAB) in intermediates and undefined starters coming from the production of different types of cheeses, while non‐starter LAB (NSLAB) display higher abundances in ripened cheeses, with the exception of spoiled products. Such separation is even more evident in the network shown in Fig. 2B, where long‐ripened (> 30 days) clustered apart from fresh/mid‐ripened cheeses and intermediates of production (natural whey cultures, fermented curds).

Interesting and relatively new application in the amplicon‐based HTS is its use for the monitoring of microbial populations beyond the species level. Genes with high polymorphism within a species can be selected for this application, potentially allowing discrimination between different strains within a species (Ercolini *et al*., 2013). In spite of the limitations due to possible sequencing errors, this application was recently exploited for monitoring *Streptococcus thermophilus* in whey and milk cultures and in cheeses, using lactose permease (*lacS*, De Filippis *et al*., 2014) and phosphoserine phosphatase (*serB*, Parente *et al*., 2016b; Ricciardi *et al*., 2016) as species‐specific and polymorphic target genes.

## Exploring microbial functions directly in food

**Figure 3:**
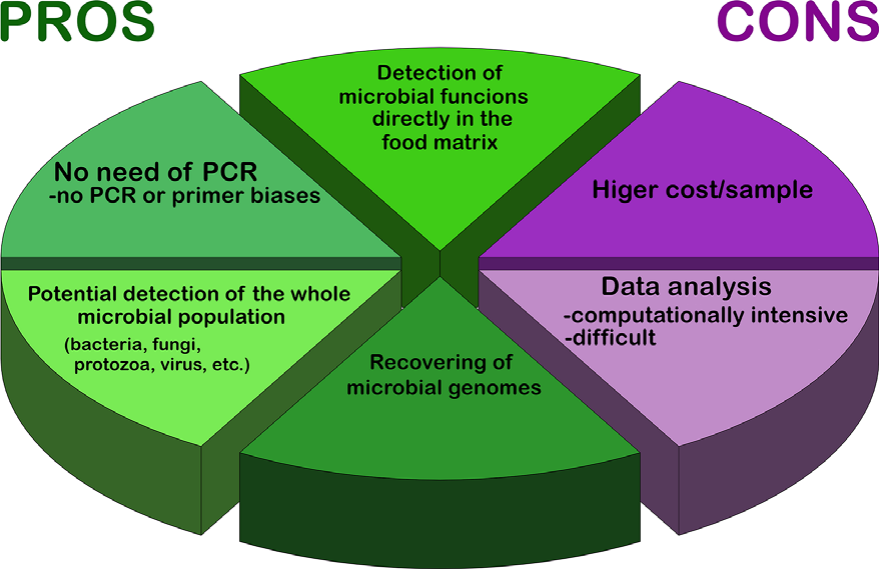
Pie chart showing pros and cons in the use of shotgun metagenomics and metatranscriptomics compared to the amplicon‐based approach.

Shotgun metagenome or metatranscriptome sequencing offers several advantages over amplicon‐based approach (Fig. 3). Whole genomes are sequenced directly (often after fragmentation and library preparation), without any prior PCR step, avoiding the possibility of amplification biases. Moreover, a picture of the entire microbial community can be obtained, tracking and comparing the abundance of bacteria and other organisms at the same time, although the methods have to be chosen carefully, in order to avoid preferential nucleic acids extractions from bacterial cells. The exceptional advantage of shotgun sequencing is the possibility to monitor the abundance of microbial activities directly in the food matrix, and hence to collect information on the genetic capacity of the whole microbial community. Moreover, it is possible to recover complete or draft microbial genomes from the metagenomes, achieving in this way a strain‐level resolution. Although metagenome and metatranscriptome sequencing potentially give a much higher amount of information their cost is still substantially higher compared with the amplicon‐based approaches. Additional technical precautions are needed in metatranscriptomics studies. For example, when sequencing messenger RNA (mRNA), all efforts are needed in order to ‘freeze’ the gene expression at sampling and protect mRNA from degradation by using stabilizing solutions, since microbial mRNA has extremely short half‐life (Rauhut and Klug, 1999; Deutscher, 2006). In addition, mRNA is only a small percentage of the total RNA extracted from the sample, which is mainly rRNA. Therefore, a further step is usually necessary, in order to reduce the amount of rRNA by bacterial or eukaryotic rRNA depletion. Finally, the bioinformatics analysis of shotgun data is much more computationally intensive and complex compared with amplicon sequencing, since well‐defined pipelines have not been implemented yet.

The above reasons have limited the application of metagenomics and metatranscriptomics to only few pioneer studies, again mainly focused on dairies. Insights about microbial activities involved in cheese ripening process were provided. Monitoring of microbial gene expression during ripening of a Camembert‐type (Lessard *et al*., 2014) and a surface‐ripened cheese (Dugat‐Bony *et al*., 2015) by metatranscriptomics revealed the presence of microbial genes involved in flavour production from amino acids and highlighted that these activities were enhanced in the first phase of ripening. Moreover, Monnet *et al*. (2016) reported differences in amino acid catabolism between the two yeasts inoculated on a Reblochon‐type cheese: *Geotrichum candidum* reached a maximum expression of amino acids‐related genes in the first phase of ripening, while *Debaryomyces hansenii* took over after 30 days, highlighting a different role of these yeasts in flavour production. Indeed, Wolfe *et al*. (2014) suggested that pathways leading to the production of flavouring compounds, such as those involved in branched‐chain and sulfur‐containing amino acid degradation, were enriched in washed‐rind compared with natural‐ and bloomy‐rind cheeses metagenomes. These studies provided an in‐depth analysis of the cheese maturation process and allowed to better understand the metabolic activities of the different community members and their possible interactions. Indeed, cheese microbial communities were proposed as simplified model to study both microbial assembly and metabolism (as affected by abiotic factors), providing a possible interesting model for understanding microbial dynamics in more complex environments (Wolfe and Dutton, 2015). In a recent example, the evolution of bacterial activities during manufacturing and ripening of a traditional cheese made with an undefined starter was monitored by metatranscriptomics highlighting how to manipulate microbial gene expression through modifications of the process parameters (De Filippis *et al*., 2016a). An increase in ripening temperature was shown to foster NSLAB growth and the expression levels of genes related to proteolysis, lipolysis and to the production of flavours from amino acids, thus promoting cheese ripening. These kinds of studies help to shed light on microbial community dynamics involved in fermentative processes and suggest how microbiome responses can be modulated in order to optimize production efficiency and product quality.

## Targeting microbial genomic variability for food fermentation processes

Technologically relevant microbial species and strains are extensively used alone or in mixed cultures in industrial processes. While allowing a better control and a standardization of the process, the use of selected cultures can lead to a loss of microbial diversity and flattening of the sensorial properties of the final products. Recent advances in microbial genomics are making it possible to exploit the wealth of genetic and phenotypic variability existing among food‐fermenting microbes (Smid and Hugenholtz, 2010). Thanks to the reduction in sequencing costs, whole genome sequencing of several strains from the same species is now a common practice. Pangenomics (the pool of essentially different genes found within a species) of common food‐borne microbes highlighted important genetic differences, which can be taken into account in order to select the best combination of species/strains for microbial industrial starters. Moreover, recently developed bioinformatics tools consent to recover microbial genomes directly from metagenomes, allowing strain‐level monitoring during the process and genomic comparison (Eren *et al*., 2015; Scholz *et al*., 2016). Comparing the genomes of five *Lactococcus lactis* strains (two wild‐type of non‐dairy origin and three dairy isolates), only about 60% of the genes were found to be shared among them and hundreds of accessory genes were not present in the dairy isolates (Siezen *et al*., 2008). Some of these coded for industrial‐interesting functions, such as exopolysaccharide (EPS) production, sugar and amino acids metabolism.

Pangenomics may also help to understand microbial evolution and adaptive mechanisms to different food niches. The genome of a *Lactobacillus delbrueckii* sub. *bulgaricus* strain industrially used for yoghurt production was compared with other collection strains of the same subspecies and genomic traits conferring higher efficiency in industrial fermentations were highlighted (Hao *et al*., 2011). The industrial strain showed higher efficiency in EPS production and well‐equipped stress tolerance, explaining its initial selection for yoghurt production. Moreover, its effective proteolytic system clarified the protocooperation mechanism with *Strepococcus thermophilus* (Hao *et al*., 2011).

Microbial interactions may be also elucidated. Coupling metagenomics and pangenomics, Erkus *et al*. (2013) characterized an undefined cheese starter culture with a long history of use. Although only two species were detected, high level of diversity at strain level was found and genome comparison suggested that a kill‐the‐winner mechanism subsisted: the phage sensitivity of the fittest strain was density‐dependent, preventing the eradication of other genetic lineages during back‐slopping regimes.

Comparative genomics of industrial strains is providing a richer and deeper understanding of the genetic composition and variability in these important microbes, promising to rapidly identify genetic *loci* that shape industrially significant traits. This will enable the development of a genetic catalogue of strains, tailoring starter composition at strain level to meet specific demands.

## Conclusions

Food fermentations are often complex *phenomena*, involving several microbial species and strains. We have learned how to identify and quantify them as well as to study their traits on the bases of state‐of‐the‐art methodologies. As discussed, the most widely used application in food microbiology is the use of amplicon‐based sequencing, leading to an in‐depth description of the ecosystem studied. This can be undoubtedly useful in order to understand microbial dynamics and evolution during food production, as well as to identify the presence of possible spoilers. Nevertheless, the real advance led by HTS is the application of shotgun metagenomics and metatranscriptomics. These approaches are still underexploited in food microbial ecology. Their application to food fermentations may be extremely useful in order to explore microbial functions directly in the food matrix and understand microbial behaviour in response to different process conditions. Moreover, recovering microbial genomes from the metagenomes allows to monitor the evolution of different strains during the process and to compare their genomic potential. These tools promise to be an invaluable help to better understand and possibly tune microbial activities in order to ensure process efficiency, product quality and safety.

## Conflicts of interest

None declared.
